# Identification, classification, and stress-responsive regulation of *HAK* family genes in poplar

**DOI:** 10.3389/fpls.2025.1690537

**Published:** 2025-11-14

**Authors:** Xiaojiao Liu, Lincui Shi, Yunyan Chen, Jing Wang, Aizhong Liu, Ping Li

**Affiliations:** 1 Key Laboratory for Forest Resource Conservation and Utilization in the Southwest Mountains of China (Ministry of Education), College of Forestry, Southwest Forestry University, Kunming, China; 2 Key Laboratory for Conservation and Utilization of in Forest Resource of Yunnan, Southwest Forestry University, Kunming, China

**Keywords:** HAK, Populus, potassium transport, stress response, phosphorylation

## Abstract

Potassium (K^+^) is essential for plant growth and high-affinity K^+^ transporters (HAKs) play vital roles in K^+^ uptake, translocation, and stress response. Although *HAK* genes have been characterized in various plants, they remain unexplored in *Populus yunnanensis*, an ecologically and economically important tree species in Southwest China. Here, we identified 32 HAKs in *P. yunnanensis* and classified them into six distinct phylogenetic groups, a structure conserved across six analyzed *Populus* species. Evolutionary analysis suggested that purifying selection (Ka/Ks < 1) has shaped all HAKs of the six tested poplar species with gene duplication events contributing to its expansion. All PyHAKs that were conserved contained abundant helical structures and transmembrane segments, which supported their conserved transport function. However, variations in protein and gene structure suggest potential functional diversification. Promoter analysis revealed an abundance of hormone-responsive cis-elements, and expression profiling confirmed that selected PyHAKs respond significantly to ABA, drought, heat, and osmotic stress. Furthermore, protein-protein interaction predictions, which were partially validated by yeast two-hybrid assays, indicated that PyHAK activity may be post translationally regulated via phosphorylation by calcineurin B-like (CBL) proteins. Our study provides the first comprehensive genomic and functional analysis of the HAK family in *P. yunnanensis*, establishing a foundation for future research on potassium regulation and stress resistance in woody plants.

## 1 Introduction

As an essential macronutrient, potassium constitutes a significant proportion of plant dry weight and plays a role in cell composition and osmotic pressure regulation (Evans and Sorger, 1966; Garcia and Zimmermann, 2014). In addition, potassium (K^+^) functions as a crucial osmotic substance whose concentration influences the activity of enzymes and the stability of protein synthesis (Li et al., 2018). In plants, roots, which serve as critical acquisition and transport tissues, are influenced by K^+^, which affects root growth, system architecture, and cellular functions (Sustr et al., 2019). The membrane transport of K^+^ is regulated by channels and transporters. Three key K^+^ transporters include K^+^ uptake permeases (KT/HAK/KUP), K^+^ transporters (Trk/HKT) and K^+^ cation proton antiporters (CPAs) (Gierth and Mäser, 2007).

The HAK/KUP/KT proteins belong to the acid polyamine organocation(APC) subfamily, which is important for K^+^ transport across membranes (Li et al., 2018). During plant development, HAK/KUP/KT proteins play important roles. Mutations in shy3-1 and KT2/KUP2 (a missense mutation) lead to reduced growth in *Arabidopsis* hypocotyls, leaves, and flowering stems, indicating the involvement of HAK/KUP/KT proteins in growth regulation (Elumalai et al., 2002). AtKT/KUP proteins influence K^+^ concentration and root hair development in *Arabidopsis* (Ahn et al., 2004). Foxtail millet SiHAK1 is involved in the regulation of K^+^ homeostasis in *Setaria italica* under K^+^ deficiency and salt stress (Zhang et al., 2018). *MeHAK5*, a positive regulator of *Arabidopsis* salt stress tolerance, exhibits high-affinity K^+^ ability and improved K^+^/Na^+^ homeostasis under K^+^ starvation conditions (Luo et al., 2024). Under K^+^ deprivation, *AtHAK5* exhibits high-affinity K^+^ and improved K^+^ uptake in *Arabidopsis* roots (Gierth et al., 2005). *OsHAK2* is involved in Na^+^ absorption and increases shoot length under low Na^+^ and low- K^+^ conditions, which also increases plant salt sensitivity (Morita et al., 2023). In natural plants with *ZmHAK4* loss-of-function mutations, increased shoot Na^+^ content is observed, differing from the function of ZmHKT1, thus revealing the functional differentiation and variation within the HAK family during maize salt tolerance (Zhang et al., 2019). Allogeneic overexpression of *CeqHAK6* and *CeqHAK11* in *Arabidopsis* increases the salt tolerance of plants by enhancing the K^+^/Na^+^ ratio and antioxidant enzyme activities and promoting germination and root growth (Wang et al., 2022).

In plants, the KT/KUP/HAK family exhibits varies among different species. Thirteen KT/KUP proteins have been identified in *Arabidopsis* (Ahn et al., 2004). Twenty-seven *O. sativa* HAK potassium transporters are clustered into six groups (Yang et al., 2009). Twenty-nine HAK/KUP/KT proteins of *S. italica* are classified into five clusters (Zhang et al., 2018). Thirty HAK/KUP/KT proteins identified in *Saccharum* sp*ontaneum* are grouped into four clusters (Feng et al., 2020). Fifty-six wheat HAK/KUP/KT strains are grouped into four clusters (Cheng et al., 2018). The 40 *Brassica napus HAK* genes are divided into four groups on the basis of phylogenetic analysis (Zhou et al., 2020). Twenty K^+^ transporters in pear (*Pyrus bretschneideri*) are grouped into three major clusters (Groups I-III) (Wang et al., 2018). Twenty-two KT/HAK/KUP transporters have been identified in purple osier willow (Liang et al., 2020). Twenty-seven barley HAK/KUP/KT proteins could be phylogenetically classified into four clusters (Cai et al., 2021). Evolutionary analysis of HAK/KUP/KT sequences from 46 plant species revealed five major groups among angiosperms (Nieves-Cordones et al., 2016). *P. yunnanensis* is a valuable poplar species native to Southwest China (Liu et al., 2022). However, research on HAKs in *P. yunnanensis* and other poplar species remains limited.

In this study, we identified 32 HAKs from *P. yunnanensis* through local BLAST searches using *O. sativa* HAKs as queries. We confirmed the PyHAKs via domain and sequence analysis. The physicochemical properties, phylogenetic relationships, conserved domains, gene structure, and cis-elements of PyHAKs were analyzed. The phylogenetic relationships of HAKs in different poplar species were analyzed. The chromosome location and collinearity analysis of poplar HAKs provided insights into the origin and expansion of these genes. To explore the regulation and function of PyHAKs, we employed cis-element analysis and qRT-PCR techniques. The interacting proteins predicted via STRING provided insights into the mechanisms of PyHAK activity, which were verified through Y2H.

## 2 Materials and methods

### 2.1 Plant material and treatment

*P. yunnanensis* was planted in the greenhouse of Southwest Forestry University under a 16-h light/8-h dark photoperiod with natural light, at a temperature of 20-25 °C. Two-month-old cuttings were transplanted into pots containing a mixture of humus soil, quartz sand, and perlite at a 3:1:1 ratio and subjected to various stress treatments, including salt stress (150 mM NaCl, 1 day), osmotic stress (25% D-mannitol, 1 day), ABA treatment (50 μM ABA, 1 day), drought stress (unwatered, 2 days), high-temperature stress (45 °C, 1 day), and low-temperature stress (4 °C, 1 day). After treatment, young leaves were collected from three individual plants per treatment (three biological replicates), immediately frozen in liquid nitrogen, and stored at -80 °C for RNA extraction. The transcriptome data utilized in this study were obtained from our previous investigation of *P. yunnanensis* under salt stress, and are publicly available in the NCBI Sequence Read Archive under the accession number PRJNA1222559. The leaf samples used for sequencing were collected from plants subjected to the following treatments: i) untreated controls (CK); ii) short-term low-concentration salt stress (25 mM NaCl for 2 days, T1); and iii) long-term high-concentration salt stress (75 mM NaCl for 2 days, T4). Gene expression levels were calculated as Fragments Per Kilobase of transcript per Million mapped reads (FPKM) values. An expression heatmap was subsequently generated via TBtools software on the basis of log2-transformed FPKM values (Li et al., 2023).

### 2.2 Identification and physicochemical analysis of *P. yunnanensis* HAK proteins

The *P. yunnanensis* HAK candidate proteins were obtained using local BLASTP (E-value: 1e-5, version: blast-2.14.1+, 10 April, 2024) with 27 rice HAKs in the *P. yunnanensis* genome (Yang et al., 2009; Sang et al., 2022). To ensure the HAK, the candidate HAK proteins were analyzed via the SMART website (http://smart.embl.de/, Apr 8, 2024) and Batch CD-Search (https://www.ncbi.nlm.nih.gov/Structure/cdd/wrpsb.cgi, Apr 8, 2024) in NCBI with conserved domains (Feng et al., 2020).

The physicochemical properties of the PyHAK proteins were analyzed via the ExPASy website (https://www.expasy.org/, Apr 8, 2024). The subcellular localization of PyHAKs was predicted via the WoLF PSORT website (https://wolfpsort.hgc.jp/, Apr 8, 2024). The prediction of transmembrane helices in PyHAKs was performed in TMHMM-2.0 (https://services.healthtech.dtu.dk/services/TMHMM-2.0/, Dec 6, 2024).

### 2.3 Phylogenetic analysis and sequence alignment of PyHAK proteins

The phylogenetic tree of PyHAKs, rice HAKs, and *Arabidopsis* HAKs was constructed with IQ-TREE (version 1.6.12 for Linux 64-bit built Mar 23, 2020) after protein sequence alignment via muscle (version 5.1, Linux64, built May 16, 2023). One HAK from green alga (*C. reinhardtii*) obtained from Phytozome (https://phytozome-next.jgi.doe.gov/, Cre17.g714200_4532) was used as an outgroup. To explore the phylogenetic relationships between PyHAKs and poplar HAKs, phylogenetic trees were constructed with PyHAK and all poplar HAK protein sequences via the maximum likelihood method (with a bootstrap value of 1000) of MEGA11 software (version 11.0.9). Finally, ITOL tool (https://itol.embl.de/) was used to enhance the visualization of the evolutionary tree.

We performed multiple sequence alignment of the functional domains via BioEdit software (version 7.0.9.0) with the whole protein sequences of PyHAKs. The reference 3D structure model of PyHAK was constructed in SWISS-MODEL (https://swissmodel.expasy.org/interactive/, May 20, 2024) with protein sequences. The aligned sequences were visualized with ESPript 3.0 (https://espript.ibcp.fr/ESPript/cgi-bin/ESPript.cgi, May 20, 2024) (Robert and Gouet, 2014).

### 2.4 Analysis of motifs, domains, gene structure and cis-elements of PyHAKs

The conserved motifs of PyHAK proteins were predicted via the MEME website (https://meme-suite.org, version 5.5.5, Apr 15, 2024) with the maximum number of motifs set to 15 and the optimum motif width set to 6-50 amino acids. The conserved domains were predicted via the Conserved Domain Database of NCBI (CDD; https://www.ncbi.nlm.nih.gov/Structure/cdd/wrpsb.cgi, Apr 15, 2024). Gene structure was analyzed via the genome annotation data of *P. yunnanensis* with the BioSequence structure illustrator package of TBtools (Toolbox for Biologists v2.142) (Chen et al., 2023). To predict the regulatory factors of *PyHAK* genes, we performed cis-acting element analysis using the promoter region as the 2000 bp genomic sequence upstream of the transcription start site of *PyHAK* coding genes. We initially extracted the upstream 2Kb sequences of *PyHAK* genes via TBtools with genome sequences and GFF3 file of *P. yunnanensis* (TBtools version 2.083) (Chen et al., 2023). Cis-acting elements were then identified via the PlantCARE website (https://bioinformatics.psb.ugent.be/webtools/plantcare/html/, Apr 15, 2024). Finally, the identified *PyHAK* cis-elements were the visualized via TBtools.

### 2.5 Chromosomal localization and collinearity analysis of *PyHAK* genes

The chromosomal localization of *PyHAK* genes was generated via the “Amazing Gene Location From GTF/GFF” package of TBtools with *P. yunnanensis* genome gff3 file. The inte raspecies collinearity analysis of *PyHAK* genes was subsequently conducted via the “Text Merger for MCScanX” tool of TBtools with the default parameters. Finally, the visualization of collinearity of *PyHAK* genes was achieved via the “circle gene view” package of TBtools (Chen et al., 2023).

### 2.6 *P. yunnanensis* RNA extraction and qRT-PCR of *PyHAK* genes

Total RNA from *P. yunnanensis* was extracted via the RNAprep Pure Polysaccharide Polyphenol Plant Total Extraction Kit (TIANGEN, DP190813, Beijing, China), with 0.1 g *P. yunnanensis* frozen leaves. The integrity and purity of the extracted RNA were evaluated via Nanodrop microvolume spectrophotometers (Thermo Scientific, USA). The All-in-One First-Strand cDNA Synthesis SuperMix for PCR Kit (TIANGEN, AT321, Beijing, China) was utilized for cDNA synthesis with total RNA. The expression levels of representative *PyHAK* genes under various stress conditions were analyzed via qRT-PCR (Bio-Rad CFX96, America). The stability of the reference gene (EF1) was confirmed across all treatment samples prior to analysis (Li et al., 2023).

### 2.7 Prediction of interaction proteins of PyHAKs

To predict the function and regulatory mechanism of PyHAKs, we utilized STRING (https://cn.string-db.org/cgi/input.pl, Apr 22, 2024) to predict the interactional proteins that interact with PyHAKs. The interacting proteins sourced from curated databases and experimentally determined, were used for interaction verification through Y2H experiments. To construct the vectors, the coding sequences of PyHAKs were subsequently cloned and inserted into pGADT7 (PT3249-5, Clontech, Japan), and coding sequences of the interacting proteins were subsequently cloned and inserted into pGBKT7 (PT3247-1, Clontech, Japan). Y2H experiments were confirmed by growth on SD/-Leu/-Trp (SD/-L-T), SD/-His/-Leu/-Trp (SD/-H-L-T), SD/–Ade/-His/-Leu/-Trp (SD/-A-H-L-T) media with different yeast concentrations (10^0^, 10^-1^and 10^-2^) and screening agent (AbA) (concentrations 0, 200, 400 and 800 μg/L) (Li et al., 2023).

## 3 Results

### 3.1 Screening and identification of *P. yunnanensis* HAK

On the basis of the sequences of 27 HAK proteins reported in rice (Yang et al., 2009), 90 candidate HAK proteins with an E value<0.05 screened in *P. yunnanensis* via local BLAST software. The candidate *P. yunnanensis* HAK proteins were verified to possess the characteristic K_trans domain (Li et al., 2018). Through Batch CD-Search Domain analysis on the NCBI website and domain screening with the pfam02705 domain on the SMART website, 32 P*. yunnanensis* HAK proteins (PyHAKs) were ultimately identified (Yang et al., 2009).

The physicochemical properties of the 32 identified *P. yunnanensis* HAK proteins were subsequently analyzed using the ExPASy tools (**Table 1**). The results revealed that the number of amino acids in the 32 HAK proteins ranged from 154 (PyHAK23) to 910 (PyHAK1), with molecular weights ranging from 17.4 kDa (PyHAK23) to 102.013 kDa (PyHAK1), and theoretical isoelectric points (pIs) ranged from 5.45 (PyHAK8) to 10.10 (PyHAK3). Two PyHAKs (PyHAK6 and PyHAK23) were shorter than the other PyHAKs and had lower molecular weights. Among all PyHAKs, 23 HAK proteins were alkaline, and the pI values of most members were mainly between 8 and 9. The grand average hydropathicity (GRAVY) of *P. yunnanensis* HAK proteins ranged from -0.650 (PyHAK3) to 0.553 (PyHAK11), whereas the aliphatic index ranged from 64.96 (PyHAK3) to 113.75 (PyHAK11). The length of the pfam02705 domain ranged from 106 (PyHAK23) to 860 (PyHAK9) amino acids, revealing the diversity of *P. yunnanensis* HAKs. Subcellular localization analysis of *P. yunnanensis* HAK proteins via the WoLF PSORT website revealed that all HAK proteins were located in plasma membrane, except for PyHAK6, which was located in the cytoplasm and had fewer amino acids. Additionally, transmembrane segments or helices were the primary characteristic and functional basis of PyHAKs, and their number varies among different PyHAK members.

**Table 1:** The physicochemical properties of PyHAK proteins.

ID	Gene name	Number of amino acids	K_trans domain	Molecular weight Da	Theoretical pI	Aliphatic index	GRAVY	Subcellular location	TMHs
Poyun00436	PyHAK1	910	157-725	102013.16	8.56	102.7	0.107	plasma membrane	12
Poyun00650	PyHAK2	824	68-638	92067.9	8.58	103.36	0.204	plasma membrane	12
Poyun00651	PyHAK3	399	64-129	44847.11	10.1	64.96	-0.65	plasma membrane	2
Poyun00653	PyHAK4	557	12-516	62033.88	8.87	108.49	0.443	plasma membrane	10
Poyun00654	PyHAK5	813	65-634	91096.21	8.84	105.83	0.258	plasma membrane	12
Poyun00774	PyHAK6	159	16-87	17678.62	7.65	88.93	0.175	cytoplasm	2
Poyun01227	PyHAK7	798	60-631	88857.85	8.09	111.94	0.354	plasma membrane	13
Poyun02111	PyHAK8	840	112-667	93268.46	5.45	105.68	0.293	plasma membrane	12
Poyun02137	PyHAK9	860	112-687	95537.12	5.56	105.49	0.279	plasma membrane	12
Poyun10325	PyHAK10	785	28-602	87244.54	8.79	111.76	0.447	plasma membrane	13
Poyun14403	PyHAK11	645	1-520	71339.08	8.73	113.75	0.553	plasma membrane	9
Poyun16214	PyHAK12	780	24-607	87505.55	8.21	108.94	0.331	plasma membrane	12
Poyun16215	PyHAK13	851	109-682	94212.74	6.12	104.21	0.336	plasma membrane	11
Poyun16282	PyHAK14	748	75-642	82634.47	7.59	107.62	0.409	plasma membrane	12
Poyun18016	PyHAK15	855	4-855	95369.79	5.68	107.02	0.275	plasma membrane	12
Poyun18865	PyHAK16	798	60-631	89766.8	8.64	107.07	0.318	plasma membrane	13
Poyun18866	PyHAK17	827	53-551	91910.24	6.16	108.13	0.228	plasma membrane	11
Poyun19116	PyHAK18	776	15-590	87202.27	9.12	104.48	0.385	plasma membrane	11
Poyun19248	PyHAK19	741	24-599	82689.84	8.63	111.65	0.447	plasma membrane	11
Poyun22962	PyHAK20	723	83-617	79820.9	7.91	103.65	0.325	plasma membrane	11
Poyun23026	PyHAK21	846	103-676	93784.42	6.14	104.81	0.324	plasma membrane	11
Poyun23027	PyHAK22	780	24-607	87364.17	8.59	107.82	0.303	plasma membrane	13
Poyun23807	PyHAK23	154	52-116	17428.29	5.51	109.42	0.313	plasma membrane	3
Poyun24811	PyHAK24	792	24-599	87858.4	6.58	108.41	0.372	plasma membrane	11
Poyun26197	PyHAK25	784	28-602	87070.5	8.98	110.55	0.466	plasma membrane	13
Poyun26324	PyHAK26	774	65-636	86867.44	8.27	103.11	0.195	plasma membrane	11
Poyun28189	PyHAK27	793	24-600	87918.3	6.63	106.42	0.365	plasma membrane	13
Poyun29646	PyHAK28	233	1-107	26523.35	8.39	109.96	0.261	plasma membrane	0
Poyun31584	PyHAK29	790	3-790	88811.53	8.73	98.81	0.251	plasma membrane	9
Poyun36717	PyHAK30	437	25-435	48702.55	9.11	103.09	0.268	plasma membrane	6
Poyun36718	PyHAK31	365	144-187	41271.62	8.85	85.4	-0.228	plasma membrane	0
Poyun38524	PyHAK32	778	46-620	86604.49	8.56	103.92	0.39	plasma membrane	12

TMHs, Transmembrane helices in proteins; GRAVY, Grand average of hydropathicity.

### 3.2 Phylogenetic analysis of PyHAKs in *P. yunnanensis* and comparison with other *Populus* species

**Figure 1:**
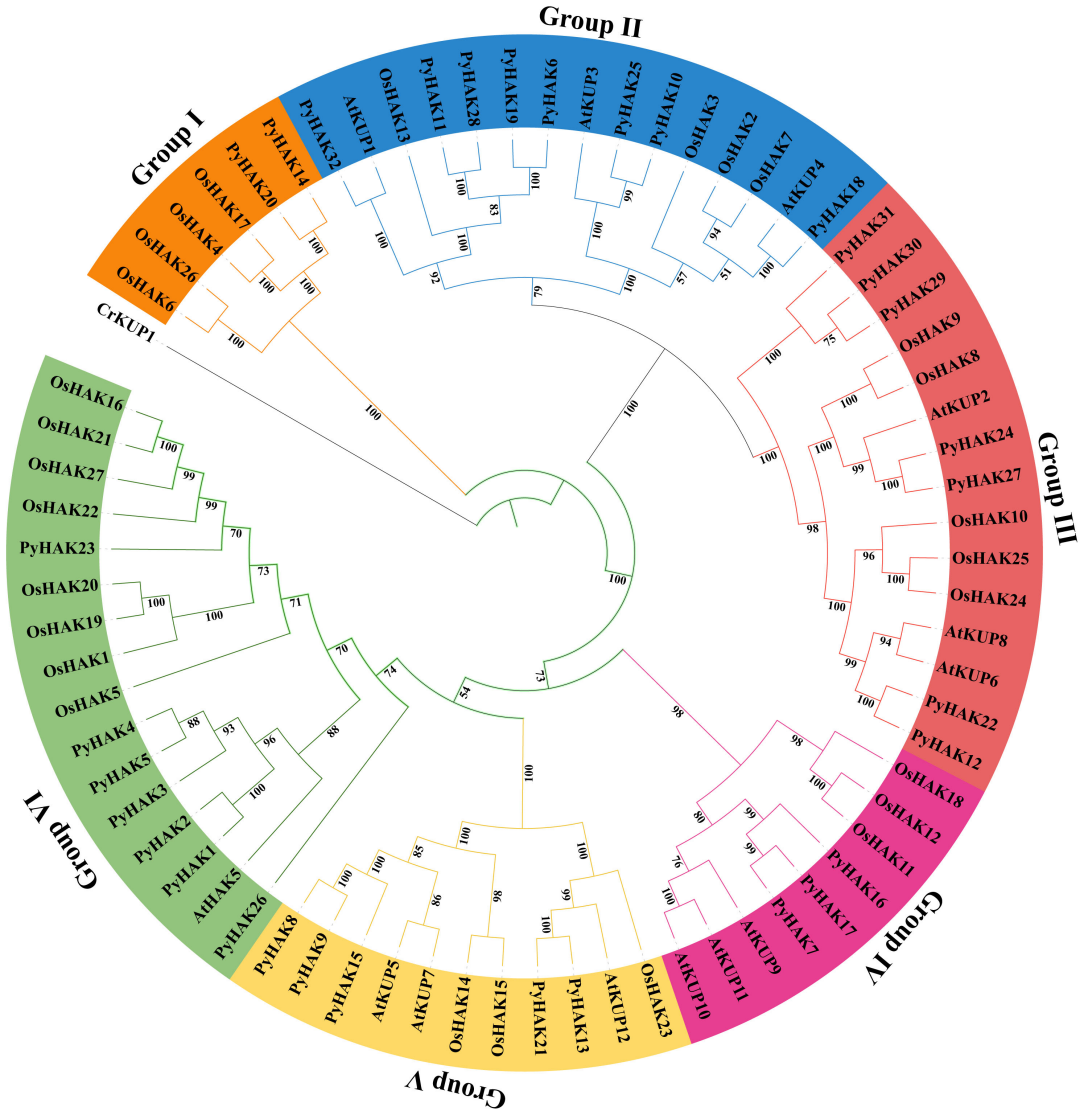
Phylogenetic tree of HAKs of *P. yunnanensis*, *O. sativa* and *Arabidopsis*. Phylogenetic tree was constructed using protein sequences of PyHAK, rice HAKs and *Arabidopsis* HKTs. CrKUP1 (One HAK from *C. reinhardtii*) was used as outgroup. Six groups were colored with different color background. Group name and cluster signs of rice and *Arabidopsis* were added. Whole protein sequences were aligned with muscle (version 5.1), and phylogenetic tree was constructed using IQ-TREE (version 1.6.12) with 1000 bootstrap values.

To explore the evolutionary relationships, we constructed a phylogenetic tree based on 32 PyHAKs, 27 rice HAKs and 13 *Arabidopsis* HAK protein sequences (**Figure 1**). In addition, one HAK from green alga (*Chlamydomonas reinhardtii*) was used as an outgroup. The results revealed that within a phylogenetic framework encompassing rice (four clusters) and *Arabidopsis*, *P. yunnanensis* HAK proteins were classified into 6 groups. The number of PyHAK members varied considerably among these different groups. Group II was the group with the largest PyHAK members (8 members, including PyHAK18, PyHAK25, PyHAK10, PyHAK32, PyHAK19, PyHAK6, PyHAK28 and PyHAK11). Both Group III and Group VI contained seven PyHAK members. Group V contained 5 PyHAK members (PyHAK21, PyHAK13, PyHAK15, PyHAK9, and PyHAK8). Group IV included 3 PyHAK members (PyHAK16, PyHAK17, and PyHAK7). Group I included 2 PyHAK members (PyHAK20 and PyHAK14).

To further investigate and compare HAKs across different poplar species, we applied the same protein selection and identification methods used for *P. yunnanensis* to five additional poplar species. We identified 49 HAKs in *Populus tomentosa*, 57 HAKs in *Populus alba*, 41 HAKs in *Populus deltoides*, 51 HAKs in *Populus euphratica*, and 28 HAKs in *Populus trichocarpa* (**Supplementary Table S1**). All poplar HAKs could be classified into the same six subgroups observed in *P. yunnanensis*, which correspond to the four established clusters in rice (**Supplementary Figure S1**). The Ka/Ks ratios between PyHAKs and their orthologs in other poplar species were predominantly less than 1, indicating the functional stability and conservation of poplar HAKs. Notably, exceptions were observed for Poyun29464 and its homologs in *P. trichocarpa*, *P. tomentosa*, *P. deltoides*, and *P. alba* (**Supplementary Table S2**).

### 3.3 The protein and gene structure analysis of PyHAKs

**Figure 2:**
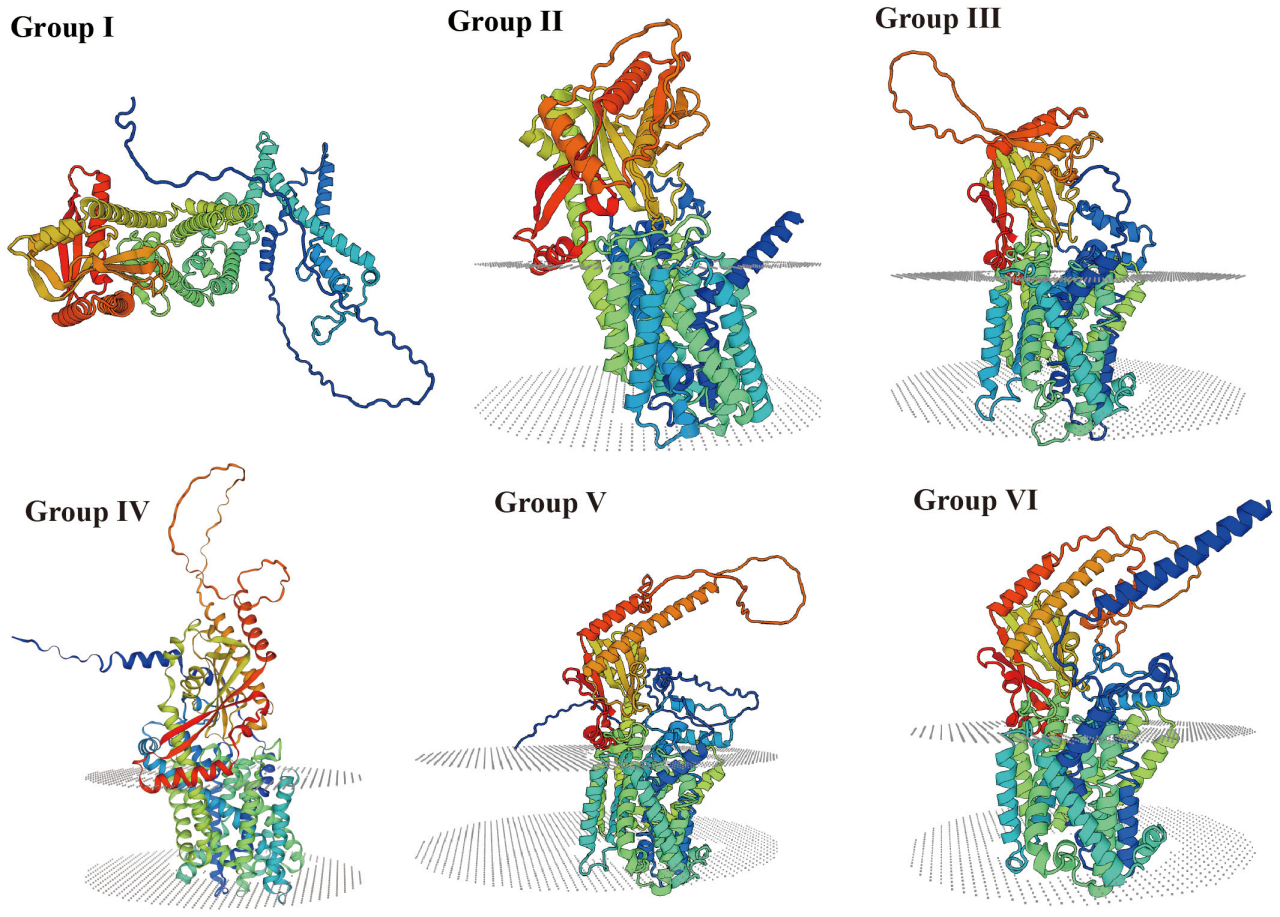
Structure and sequence alignment of PyHAKs. Group I, the 3D protein model of PyHAK20 was built based on A0A2K1ZH10.1.A (AlphaFold DB model of A0A2K1ZH10_POPTR, *P. trichocarpa*) with 0.76 GMQE and 99.31% seq identity in SWISS-MODEL. Group II, the 3D protein model of PyHAK18 was built based on A0A2K1ZH10.1.A (Potassium transporter, AlphaFold DB model of A0A2P2JHU7_RHIMU, *Rhizophora mucronata*) with 0.76 GMQE and 72.42% seq identity in SWISS-MODEL. Group III, the 3D protein model of PyHAK12 was built based on V4VGN3.1.A (Potassium transporter, AlphaFold DB model of V4VGN3_CITCL, *Citrus clementina*) with 0.77 GMQE and 86.89% seq identity in SWISS-MODEL. Group IV, the 3D protein model of PyHAK16 was built based on K7KPZ9.1.A (Potassium transporter, AlphaFold DB model of K7KPZ9_SOYBN, *Glycine max*) with 0.77 GMQE and 85.46% seq identity in SWISS-MODEL. Group V, the 3D protein model of PyHAK21 (group V) was built based on A0A1R3I6Q1.1.A (Potassium transporter, AlphaFold DB model of A0A1R3I6Q1_COCAP, *Corchorus capsularis*) with 0.73 GMQE and 83.16% seq identity in SWISS-MODEL. Group VI, the 3D protein model of PyHAK2 was built based on A0A2R6QQ77.1.A (Potassium transporter, AlphaFold DB model of A0A2R6QQ77_ACTCC, *Actinidia chinensis*) with 0.75 GMQE and 71.85% seq identity in SWISS-MODEL. The predicted transmembrane segments were added with grey lamella.

Sequence alignment of 32 P*. yunnanensis* HAK proteins revealed the presence of highly conserved K_trans functional domains across all the sequences (**Table 1**, **Supplementary Figure S2**). Additionally, these sequences were clustered according to the characteristic 3D structure models of K^+^ transport built by SWISS-MODEL and group classification (**Figure 2**). The abundant α-helix, β-strand, and connecting regions in the structures of PyHAK proteins ensure their transport function (Henderson 1993). Transmembrane segments were predicted for most PyHAKs, excluding those in Group I. Conversely, the 3D protein templates used for constructing the PyHAK model varied among different PyHAK groups, and these templates were all potassium transporters from different plant species. The diverse potassium transporter templates from different plants and their corresponding global model quality estimate (GMQE) values highlighted the differences among different HAK groups. Overall, the protein sequences of PyHAK members within the same group presented high similarity (**Supplementary Figure S2**).

**Figure 3:**
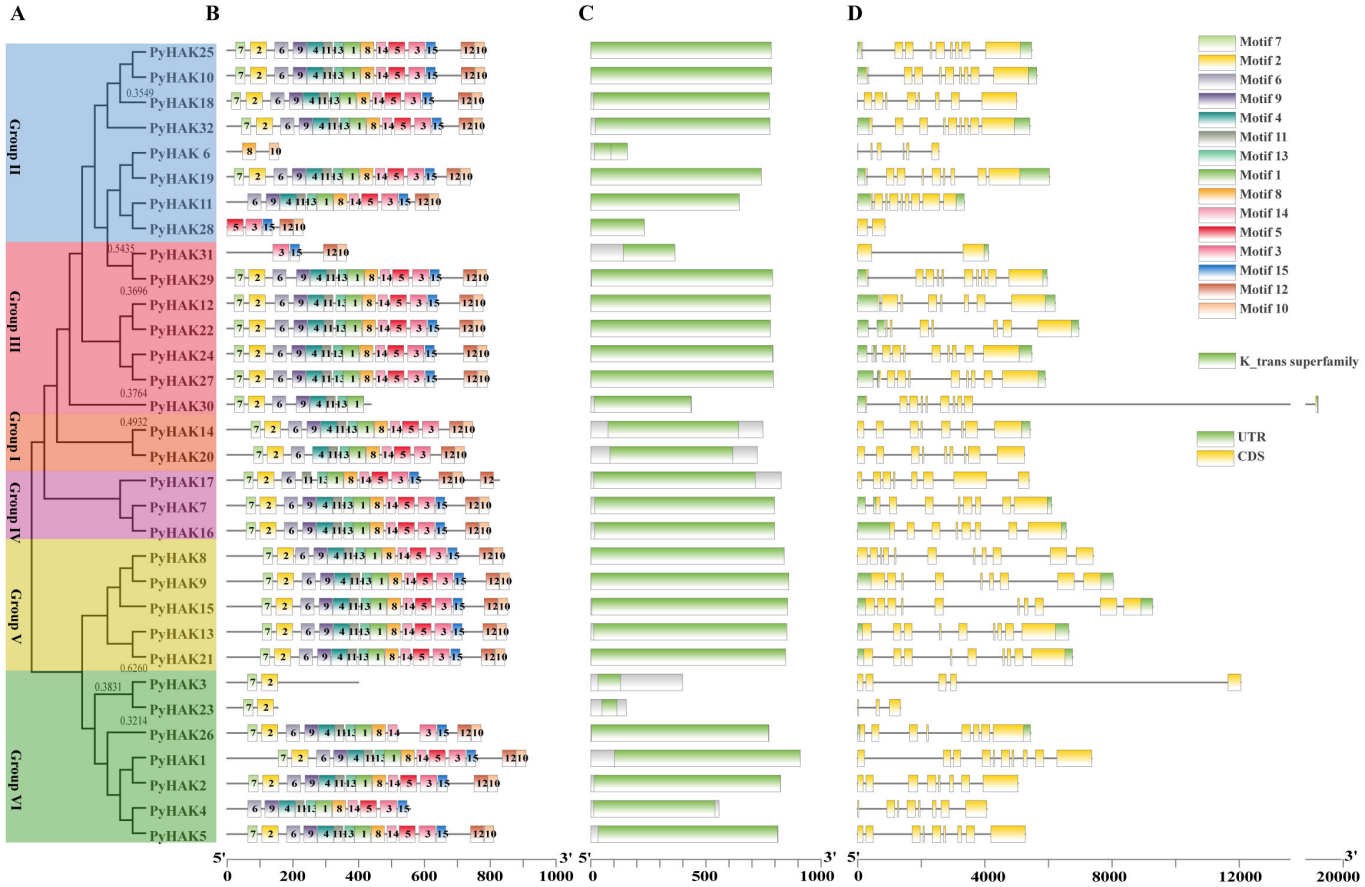
Phylogenetic tree, protein and gene structure of PyHAKs. **(A)** Phylogenetic tree of PyHAKs. The physiology tree was constructed using PyHAK protein sequences with MEGA (1000 bootstrap values). **(B)** Conserved motif analysis of PyHAKs using their protein sequences. A threshold of 15 motifs was set. **(C)** The conserved domains of PyHAKs. The K_trans superfamily represented the characteristic domain of PyHAKs. **(D)** Gene structure of *PyHAK*s. Scale lines below indicate the lengths of PyHAK proteins and genes. Group classifications are denoted by color boxes. Bootstrap values above 0.3 are indicated in the phylogenetic tree. Annotations for color boxes (motifs, domain, and gene structure) are provided in the upper right corner of the figure.

On the basis of the protein sequences of PyHAKs, six groups of PyHAKs were identified according to their evolutionary branches (**Figure 3A**). The conserved motifs also showed clustering conservation among PyHAKs. When setting the threshold was set at 15 motifs, most PyHAKs contained more than 10 conserved motifs, except for PyHAK6, PyHAK28, PyHAK31, PyHAK23, and PyHAK3 (**Figure 3B**). Both PyHAKs in group I contained all 15 motifs. The Group V PyHAKs were also conserved and contained 15 motifs. All three PyHAKs in group IV had more than 14 motifs. The number of motifs in groups II, III and VI varied due to the short length of these PyHAKs. Despite the variation in conserved motifs among PyHAKs, all members possessed conserved K+ transport domains of different lengths, which are characteristic functional domains of K+ transporters (**Figure 3C**). The gene structure of *PyHAK*s shows variations in the number of exons. Groups I, IV, and V had conserved numbers of exons, all exceeding eight. The number of exons varied among groups II, III, and VI, corresponding to their gene lengths and protein sequences (**Figure 3D**). These results indicated that PyHAKs presented conserved functional structures and varied protein and gene sequences.

### 3.4 Chromosomal localization and collinearity analysis of *PyHAK*s

To further investigate the potential positional relationships among *HAK* genes in *P. yunnanensis*, we analyzed their chromosomal positions. Chromosome mapping (LG) revealed an uneven distribution of *HAKs* across the chromosomes of *P. yunnanensis*. Specifically, chromosome LG01 harbored 9 *PyHAK*s, LG07 contained 5 *PyHAK*s, LG09 had 4 *PyHAK*s, LG06 carried 3 *PyHAK*s, and both LG11 and LG18 each contained 2 *PyHAK*s. Chromosomes LG04, LG05, LG10, LG12, LG13, LG14, and LG19 each possessed only 1 *PyHAK*.

**Figure 4:**
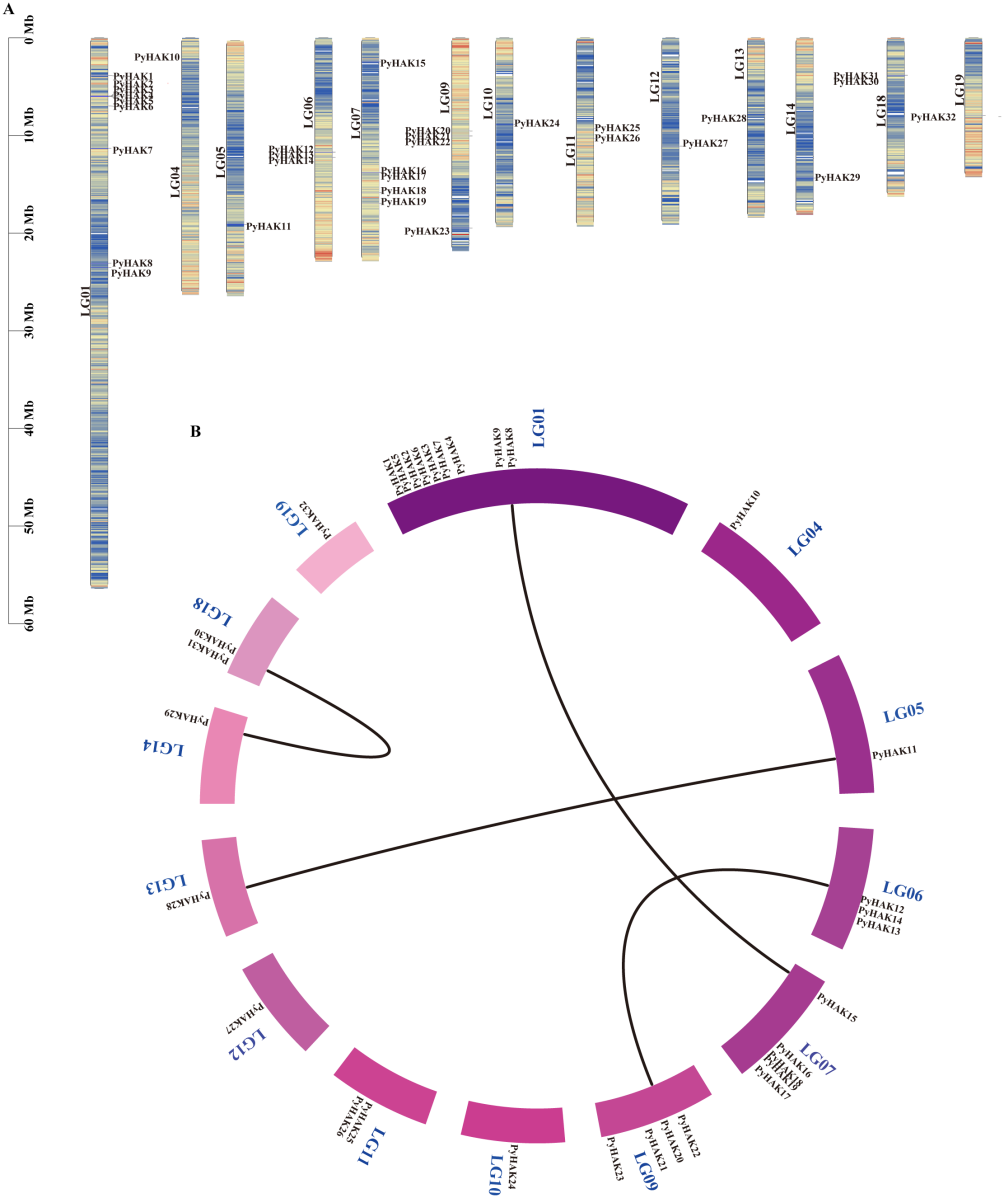
Chromosome location and collinearity analysis of *PyHAK*s. **(A)** Chromosome locations of 32 *PyHAK*s. LG means chromosome, with the chromosome numbers indicated on the left side of the chromosomes. **(B)** Collinearity analysis of 32 *PyHAK*s. The lines represented collinearity pairs in *P. yunnanensis*.

To explore the evolutionary relationships among *HAKs* in *P. yunnanensis*, we performed an intraspecific collinearity analysis of the 32 *PyHAK*s. Four pairs of tandemly duplicated genes located on different chromosomes were identified: *PyHAK8* (LG01) and *PyHAK15* (LG07), *PyHAK30* (LG18) and *PyHAK29* (LG14), *PyHAK12* (LG06) and *PyHAK20* (LG09), *PyHAK28* (LG13) and PyHAK11 (LG05) (**Figure 4B**). These pairs were mainly members of the same group, which is characteristic of segmental duplication. The collinearity analysis of HAKs across different poplar species demonstrated the tandem relationships among poplar HAKs (**Supplementary Figure S3**; **Supplementary Table S3**). A total of 35 collinearity pairs were identified between *P. alba* and *P. yunnanensis* HAKs, with group II and group III PyHAKs being the major members (19 pairs). A total of 42, 60, 30 and 40 HAK collinear pairs were identified between *P. yunnanensis* and *P. trichocarpa*, *P. yunnanensis* and *P. tomentosa*, *P. yunnanensis* and *P. euphratica*, *P. yunnanensis* and *P. deltoides*, with a large proportion of group II and III members. The collinearity pairs between *P. yunnanensis*, *O.sativa* and *Arabidopsis* also revealed the conservation and origin of HAKs in the same clusters.

### 3.5 Analysis of cis-acting elements of *HAK* genes in *P. yunnanensis*

Cis-acting elements in gene promoters play a crucial roles in elucidating transcriptional regulation and functional diversity. In this study, we performed a comprehensive analysis of cis-acting elements located within the promoter regions (2 kb before trans-start sites) of 32 P*. yunnanensis HAK* genes.

**Figure 5:**
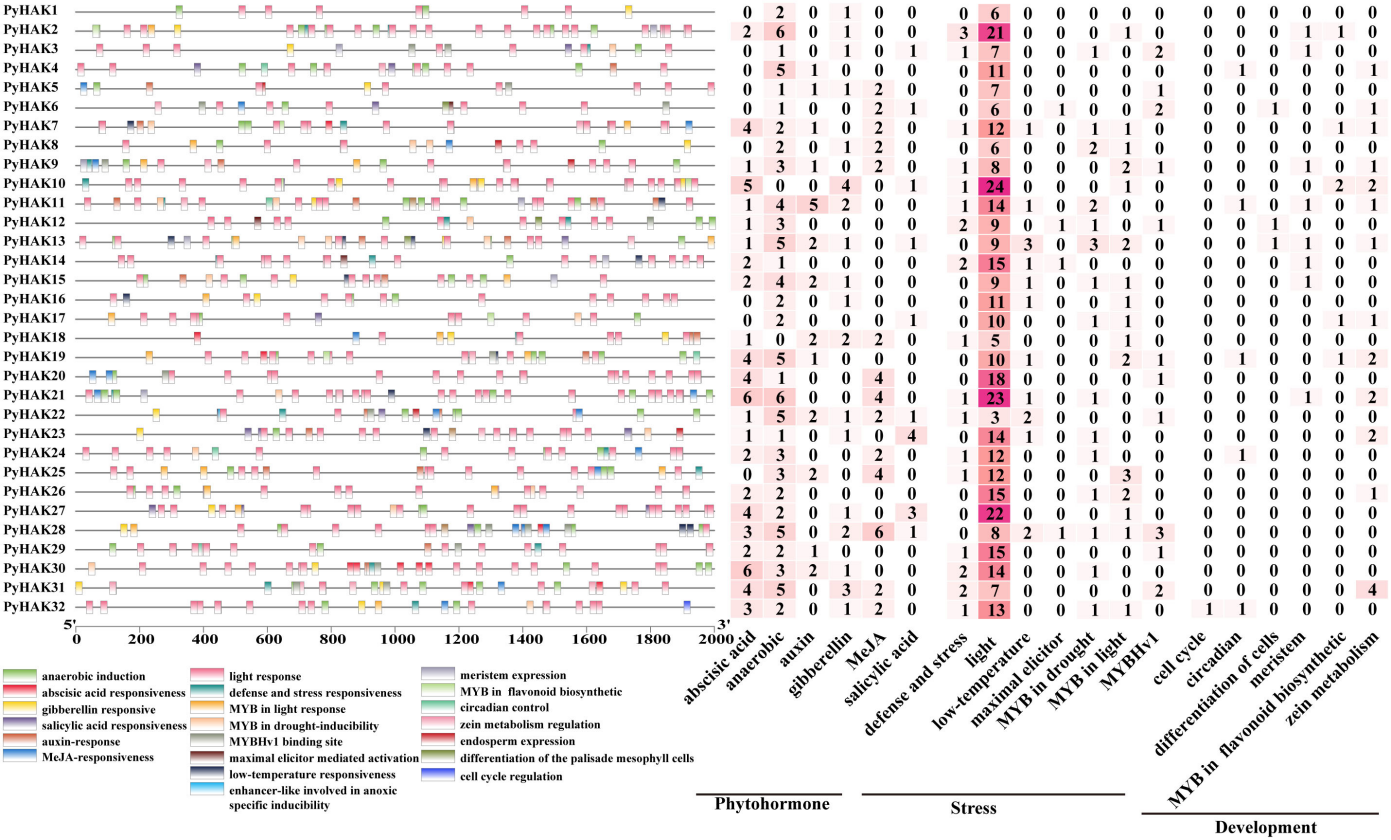
Cis-elements analysis of the promoter region of *PyHAK*s. The color boxes below reflected cis-elements identified in the promoter regions of *PyHAK*s. The numbers of the cis-elements were shown on the right.

Our investigation revealed that *PyHAK* promoters contained a diverse array of cis-acting elements associated with various biological processes and stress responses (**Figure 5**). Apart from transcription activity elements such as the TATA box and CAAT box (**Supplementary Table S4**), the majority of these elements are linked to stress responses and growth regulation (**Figure 5**). Among these, abiotic stress response elements were the most enriched in the *PyHAK* gene promoters. In addition to light response elements, those associated with defense and stress responsiveness, particularly MYB transcription factor-binding sites, were significantly enriched, highlighting the functional significance of *PyHAK*s. Phytohormone-related cis-elements, including those for ABA, GA (gibberellin), MeJA (methyl jasmonate), and SA (salicylic acid) were also present in the *PyHAK*s promoters, indicating the regulatory role of phytohormones in *PyHAK* regulation. Abiotic stress-related elements, such as those related to anoxia, low-temperature, and SA, were also enriched in *PyHAK* promoters. A few *PyHAK*s are predicted to be regulated by promoters containing elements related to plant growth development as well as metabolite synthesis, such as those associated with the cell cycle, circadian rhythm cell differentiation, meristem and zein metabolism. Notably, 20 PyHAKs contained cis-elements associated with more than four types of stress and hormone signals, particularly members of groups II and III. Some PyHAKs, such as *PyHAK22*, *PyHAK7*, *PyHAK13*and *PyHAK19*, are predicted to be coordinately regulated by multiple phytohormones and stresses. These findings provide a robust foundation for future functional studies aimed at uncovering the specific roles and interactions of *HAK*s under diverse growth and stress conditions.

### 3.6 Verification of the expression of *PyHAK* genes under stress

**Figure 6:**
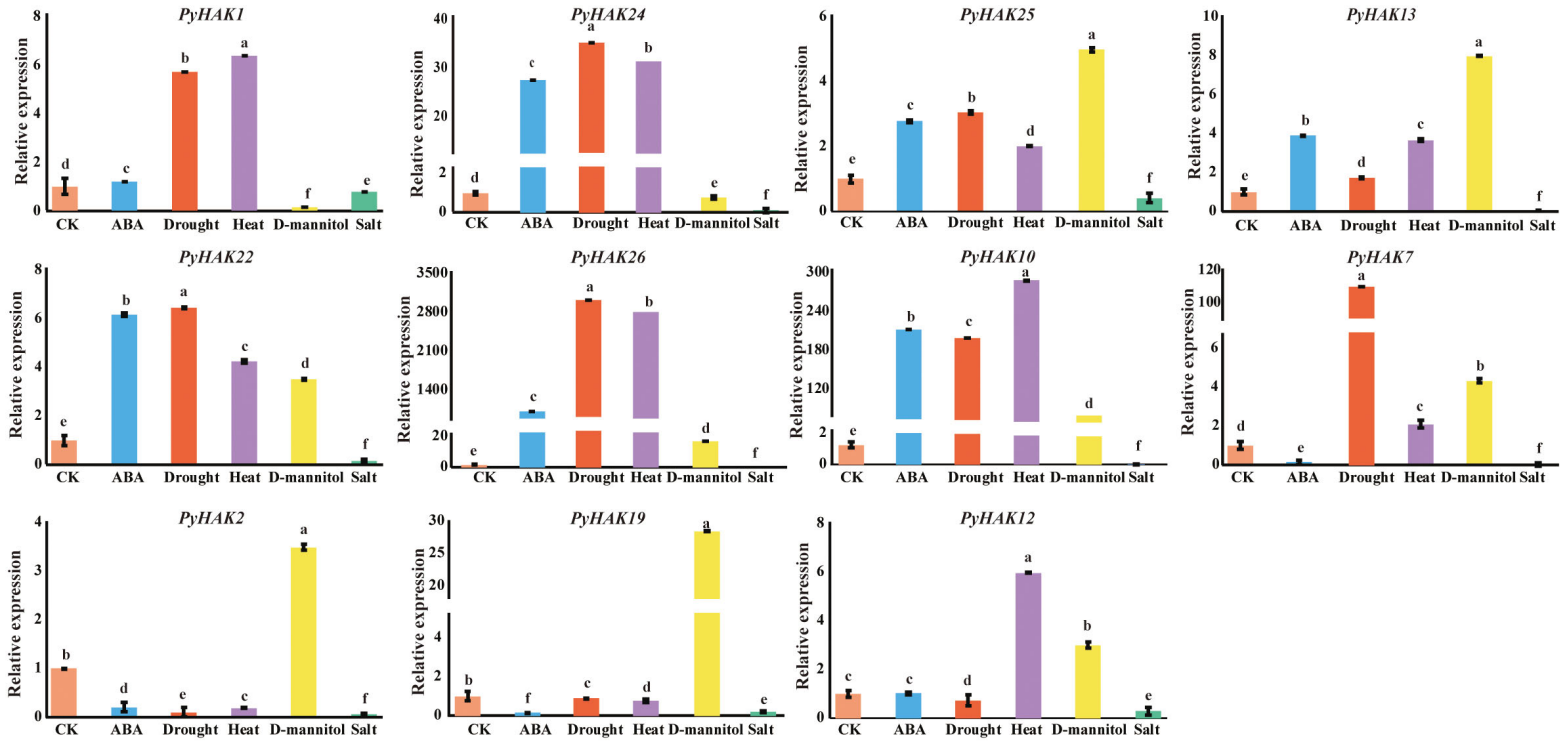
Relative expression of *PyHAK*s under stress treatment. The relative expression levels of *PyHAK*s under stress were presented using different color boxes. CK represented untreated control, ABA, drought, heat, D-mannitol and salt represented stress treatments in *P. yunnanensis*. Different letters on the top of the boxes indicated significant differences among treatments.

To verify the response of *PyHAK* genes to stress, we subjected *P. yunnanensis* to various abiotic stress treatments, including drought, heat, salt, D-mannitol and ABA treatments. Eleven representative *PyHAK* genes were selected on the basis of their specific response under salt stress (**Supplementary Figure S4**; **Supplementary Table S5**) for expression level measurement via qRT-PCR. The primers used for qRT–PCR are listed in **Supplementary Table S6**. All the tested *PyHAK* genes presented significant expression changes under stress treatment (**Figure 6**). ABA, drought, heat, and D-mannitol stress treatments induced the expression of most *PyHAK*s genes, including *PyHAK25*, *PyHAK13*, *PyHAK22*, *PyHAK26* and *PyHAK10*. The relative expression of *PyHAK1* and *PyHAK24* was induced by drought, heat, and ABA treatments. Similarly, the relative expression of *PyHAK7* was induced by drought, heat and D-mannitol stress. The relative expression of *PyHAK12* was induced by heat and D-mannitol stress. However, the relative expression of *PyHAK2* and *PyHAK19* was induced by D-mannitol treatment only. On the other hand, all *PyHAK* genes were downregulated under salt stress, which was in accordance with the known functions of K^+^ transporters under salt stress.

### 3.7 The prediction of interaction proteins of PyHAKs

**Figure 7:**
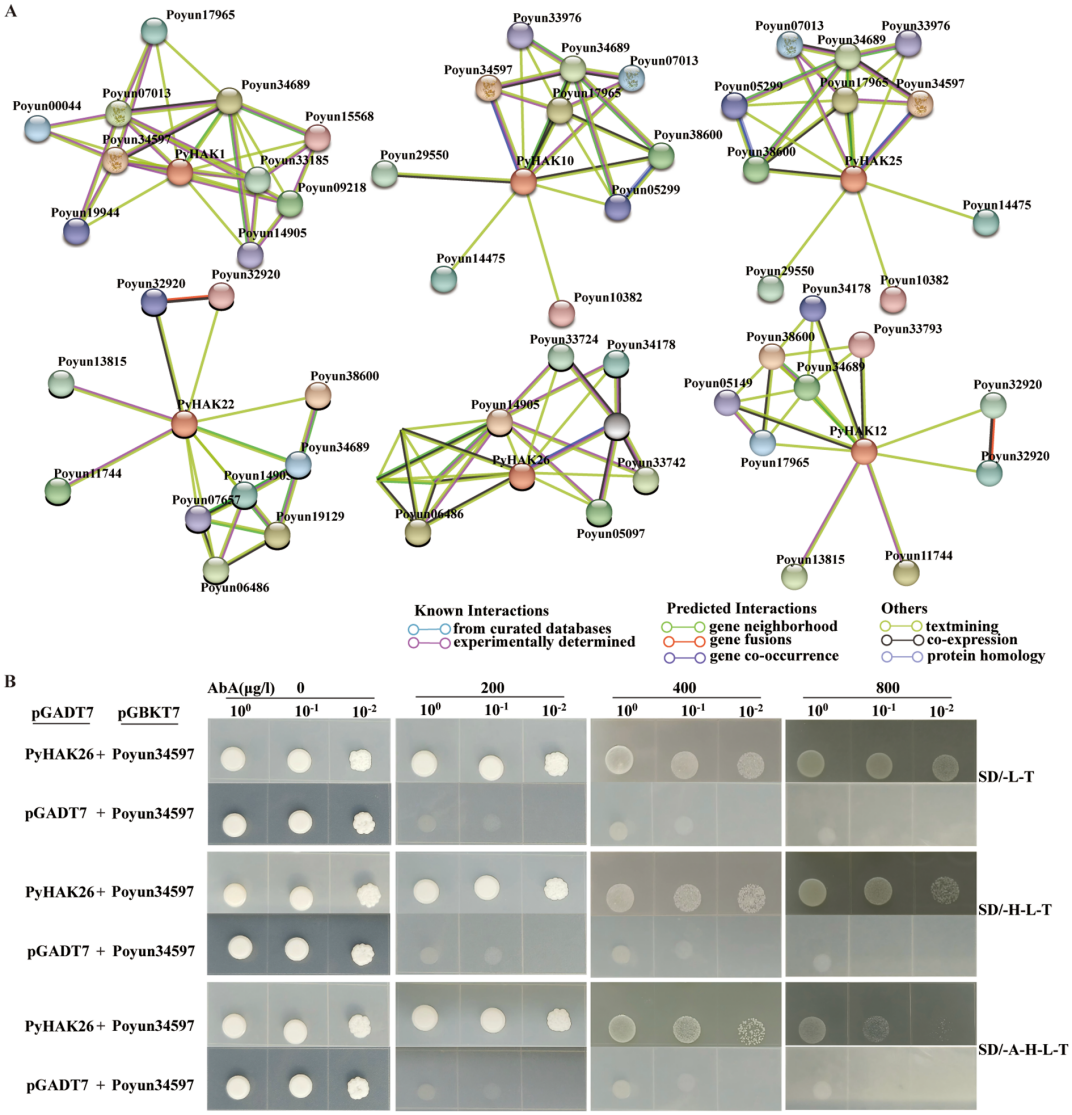
Interaction relationship prediction and verification of PyHAKs. **(A)** The interaction relationship of PyHAKs predicted using STRING. The balls represented PyHAKs and their interacted proteins. Different colored lines represented the prediction methods for interaction relationships, as explained in the lower right corner. **(B)** Protein interaction relationship verification with Y2H assay. The coding sequences of PyHAK26 (PyHAK) and Poyun34597 (CBL) were cloned into the activation domain vector (pGADT7) and GAL4 DNA binding domain vector (pGBKT7), respectively. The negative control was pGADT7 without any protein coding sequence and pGBKT7 with Poyun34597 coding sequence. SD/-L-T, SD/-H-L-T, and SD/-A-H-L-T represented SD-Leu-Trp, SD-His-Leu-Trp, and SD-Ade-His-Leu-Trp medium for yeast culture, respectively. Yeast at different concentrations was used for testing (10^0^, 10^-1^, 10^-2^). The screening agent (AbA) concentrations (0, 200, 400, 800 μg/L) were indicated on the top.

To confirm the regulatory mechanism of PyHAKs, we predicted the interacting proteins via STRING (**Figure 7A**; **Supplementary Table S7**). Six PyHAKs were predicted to interact with a complex network of proteins: PyHAK1 (group VI), PyHAK26 (group VI), PyHAK10 (group II), PyHAK25 (group II), PyHAK22 (group III) and PyHAK12 (group III). Ion transporters, including K^+^, Na^+^ and H^+^, were the common interacting proteins of PyHAKs. Moreover, in addition to transporters, K^+^ channels, calmodulin, protein kinases and AP2/ERF transcription factors were also predicted to interact with PyHAKs, particularly PyHAK1.

To further investigate the interaction between PyHAKs and their predicted interacting proteins, we constructed yeast expression vectors of PyHAKs and their predicted interacting proteins. A Y2H assay was used to verify the interaction between these proteins. Poyun34597 (CBL) interacted with PyHAK (PyHAK26) and activated the yeast reporter gene, allowing normal growth under the screening agent (Aureobasidin, AbA) at a specific dilution concentration (**Figure 7B**).

## 4 Discussion

As pivotal transporters for potassium (K^+^) uptake and homeostasis, the HAK/KUP/KT family governs essential functions in plant growth and stress adaptation (Li et al., 2018; Grabov, 2007). While extensively characterized in model plants such as *Arabidopsis* and rice (Ahn et al., 2004; Yang et al., 2009), their composition and functional landscape in perennial trees remain less explored. Our genome-wide analysis of *Populus yunnanensis*, a species of ecological and economic importance in Southwest China (Liu et al., 2022), identified 32 HAK transporters (PyHAKs). Through integrated phylogenetic, structural, and expression analyses, we not only confirmed the conserved nature of this transporter family in plant potassium homeostasis but also revealed evidence of functional diversification, particularly in response to abiotic stresses. This study provides a comprehensive framework for understanding potassium regulation in woody plants and pinpoints key genetic candidates for improving stress resistance.

The copy number of HAK transporters varies considerably across plant species, a variation that is often correlated with genome size and complexity, as exemplified by the expanded families in wheat and *B. napus* (Cheng et al., 2018; Zhou et al., 2020). Notably, this trend of lineage-specific expansion is also observed within the genus *Populus*, where differences in *HAK* gene numbers among species, including *P. yunnanensis*, *P. trichocarpa* (He et al., 2012), have distinct evolutionary histories. In this study, domain-based screening identified 32 HAK members in *P. yunnanensis* (**Table 1**). While the defining K_trans domain is a conserved hallmark of the family, we observed notable variation in its length among the PyHAKs. This divergence in the core functional domain strongly implies subsequent functional diversification within the PyHAK family (Cheng et al., 2018), potentially equipping them with specialized roles in potassium homeostasis. This phenomenon of functional specialization is well-established in model plants; for example, in *Arabidopsis*, *AtHAK1* and *AtHAK5* have diverged to mediate long-distance Na^+^ recirculation and high-affinity K^+^ uptake, respectively (Berthomieu et al., 2003; Ródenas et al., 2021).

The phylogenetic classification of PyHAKs into six distinct clades aligns with the established framework in *Arabidopsis* and rice. This six-clade system is further conserved within the genus *Populus* (**Figure 1**), as conclusively demonstrated by our analysis of *P. yunnanensis* and five other poplar species (**Supplementary Table S1**; **Supplementary Figure S1**, He et al., 2012). This consistency underscores the deep evolutionary conservation of the HAK family in woody angiosperms. We found that members within each clade share a high degree of conservation in protein motifs and gene structures, suggesting cohesive functional roles (**Supplementary Figure S2**; **Figures 2**, **3**, Cai et al., 2021; Feng et al., 2020). Conversely, pronounced disparities in these features between clades provide compelling evidence for functional diversification (Xu et al., 2012). This diversification was likely facilitated by the family’s expansion through segmental duplication events, as revealed by collinearity analysis (**Figure 5**; Yang et al., 2009). Crucially, the overwhelmingly purifying selection (Ka/Ks < 1) acting on these duplicated pairs indicates strong evolutionary pressure to maintain the core K^+^ transport function (**Supplementary Table S2**, Tajo et al., 2023), whereas the retention of multiple copies has potentially enables subfunctionalization (Cai et al., 2021; Jin et al., 2021), fine-tuning aspects such as expression patterns or regulatory mechanisms to support the complex physiology of a perennial tree.

The results of the cis-elements and expression analyses collectively confirmed the pivotal role of PyHAKs in orchestrating adaptive responses to abiotic stresses. The prevalence of hormone-responsive cis-elements (e.g., for ABA, MeJA) in PyHAK promoters (**Figure 5**, **Supplementary Table S4**) provides a mechanistic basis for their transcriptional regulation, positioning them as key nodes in stress signaling networks (Lieberman-Lazarovich et al., 2019). This finding was functionally corroborated by our qRT-PCR results, in which multiple *PyHAK*s were significantly upregulated under drought, osmotic, and heat stresses (**Figure 6**). This induction aligns with the critical function of K^+^ in maintaining cellular turgor and homeostasis under dehydrating conditions, suggesting a concerted effort by the plant to increase K^+^ uptake capacity to mitigate stress damage (Zhao et al., 2020). The differential PyHAK expression under salt stress revealed a concentration-dependent strategy. While induced at lower salinities (25-75 mM NaCl) (**Supplementary Figure S4**; **Supplementary Table S5**), which are likely to support K^+^ nutrition, most members were suppressed under severe stress (200 mM NaCl) (**Figure 6**). We propose that this downregulation is an adaptive energy-saving and protective measure. Under extreme Na^+^ pressure, sustaining high-affinity K^+^ uptake becomes metabolically costly and potentially counterproductive, as nonselective transporters could facilitate Na^+^ influx (Shabala and Cuin, 2008). Thus, suppressing specific PyHAKs may conserve ATP for essential Na^+^ extrusion (e.g., via SOS) and osmolyte synthesis while concurrently reducing Na^+^ entry (Yang and Guo, 2017). This distinct, stress-severity-dependent expression underscores the functional specialization within the PyHAK family.

In addition to their role as potassium transporters, the predicted protein-protein interaction network positions PyHAKs as central hubs within a broader signaling and regulatory framework, potentially explaining their multifaceted roles in stress adaptation (Garg and Kühn, 2022). The interaction with CBL-CIPK complexes suggests a conserved, phosphorylation-dependent mechanism for the posttranslational activation of specific PyHAKs, directly linking calcium signaling to rapid K^+^ uptake modulation under stress (**Figure 7**; **Supplementary Table S7**, Ragel et al., 2015; Batistic and Kudla, 2009). Furthermore, connections to 14-3-3 proteins imply a layer of regulatory control over protein stability and activity (Huang et al., 2021). The interplay with other ion transporters, such as the potassium channel AKT1 and abiotic stress-linked ABC transporters, points to coordinated mechanisms for fine-tuning ion homeostasis (Yang et al., 2023; Liu et al., 2025). Finally, the predicted associations with AP2/ERF transcription factors suggest a plausible pathway for the transcriptional reprogramming of downstream stress-responsive genes (Xie et al., 2019). While these computational insights require experimental validation, they generate specific, testable hypotheses regarding the posttranslational, homeostatic, and transcriptional mechanisms that underpin PyHAK-mediated stress adaptation.

## 5 Conclusions

In this study, 32 HAKs were identified in *P. yunnanensis* through homologous alignment with HAK domains. The physicochemical properties of the 32 PyHAKs varied, as did their phylogenetic clusters. The 32 PyHAKs were classified into six groups, including four clusters that corresponded to those in *Arabidopsis* and *O. sativa*. All identified poplar HAKs could be classified into the same groups and clusters, which were under positive selection. The protein structure of all PyHAKs was conserved, containing transmembrane segments and abundant helical structures, but exhibited variations in conserved motifs and domain lengths. Tandem distribution and collinearity analyses revealed intraspecific and interspecific segmental duplication events. The identification of cis-elements related to hormones and transcription factors indicated the function of PyHAKs under stress, which was verified through qRT-PCR. The interaction between PyHAK and CBL also revealed the activity of PyHAK under stress via phosphorylation.

## Data Availability

The datasets supporting the conclusions of this article are included within the article and its additional files. The poplar sequences in this article were downloaded from BIG (https://ngdc.cncb.ac.cn/) with accession number PRJCA010101. The transcriptome data used in this study are deposited in the NCBI Sequence Read Archive under the accession number PRJNA1222559.

## Supplementary material

Supplementary Figure 1 Phylogenetic trees of all poplar HAKs.

Supplementary Figure 2 Sequence alignment of PyHAKs proteins during different groups.

Supplementary Figure 3 Collinearity analysis across *P. yunnanensis*, poplar species, *O.sativa* and *Arabidopsis.*

Supplementary Figure 4 Expression patterns of *PyHAK* genes based on RNA-seq analysis.

Supplementary Table 1 Message of HAKs identified in poplar.

Supplementary Table 2 Ka/ks values calculated between HAKs across different poplar species.

Supplementary Table 3 Collinearity analysis of HAKs across plant species.

Supplementary Table 4 Cis-elements predicted in the promoter of *PyHAK*s.

Supplementary Table 5 Expression values of *PyHAK* genes for the heatmap.

Supplementary Table 6 Primers for qRT-PCR.

Supplementary Table 7 Interacted proteins of PyHAKs predicted in STRING.
