# Supplementary figures and images for: Identification, classification, and stress-responsive regulation of HAK family genes in poplar
Source: Front Plant Sci. 2025 Nov 14;16:1690537. doi: 10.3389/fpls.2025.1690537 (PMC12660106; doi:10.3389/fpls.2025.1690537)

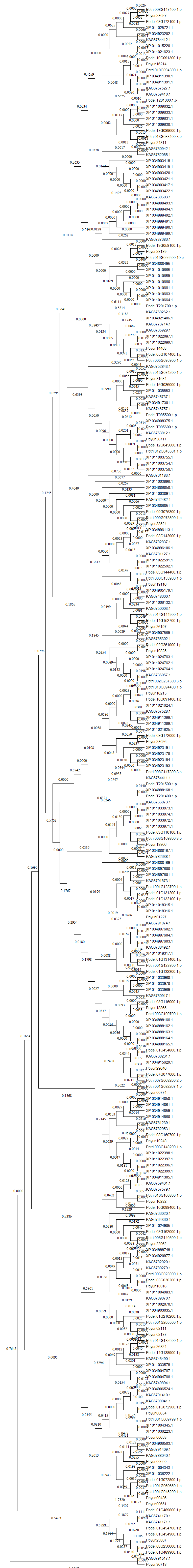

Supplement: Supplementary Figure 1 — Phylogenetic trees of all poplar HAKs. [file Image1.tif]

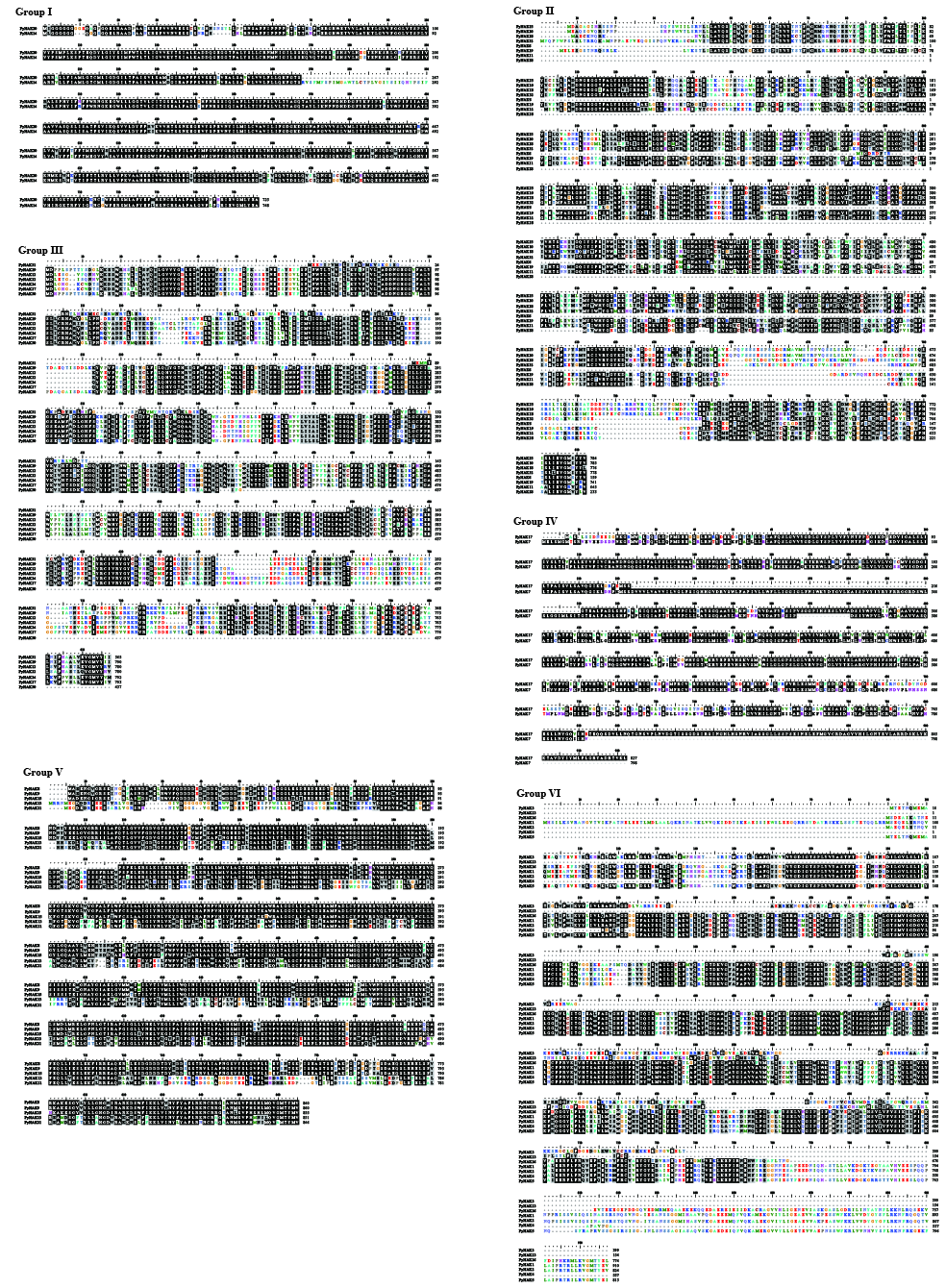

Supplement: Supplementary Figure 2 — Sequence alignment of PyHAKs proteins during different groups. [file Image2.tif]

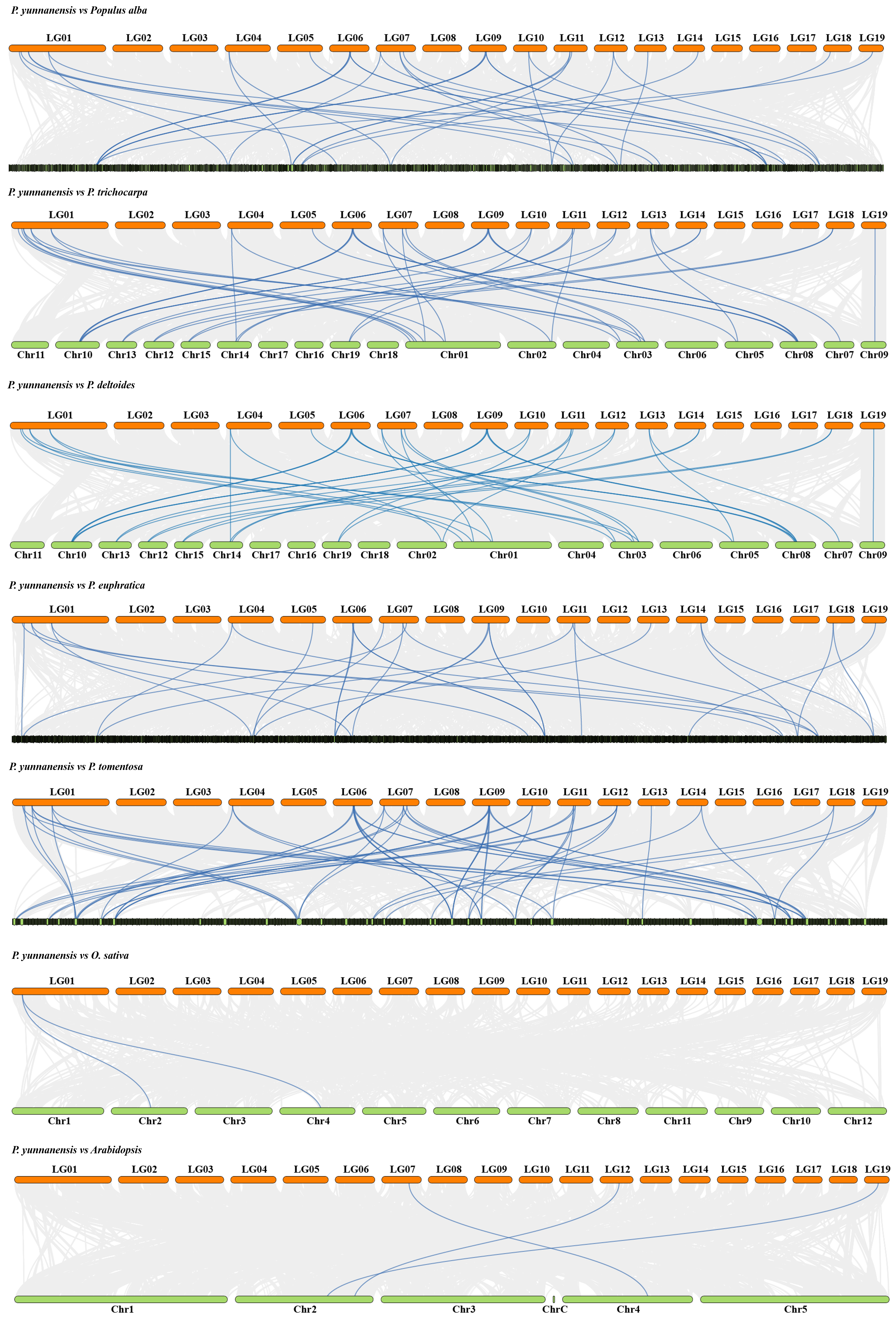

Supplement: Supplementary Figure 3 — Collinearity analysis across P. yunnanensis, poplar species, O.sativa and Arabidopsis. [file Image3.tif]

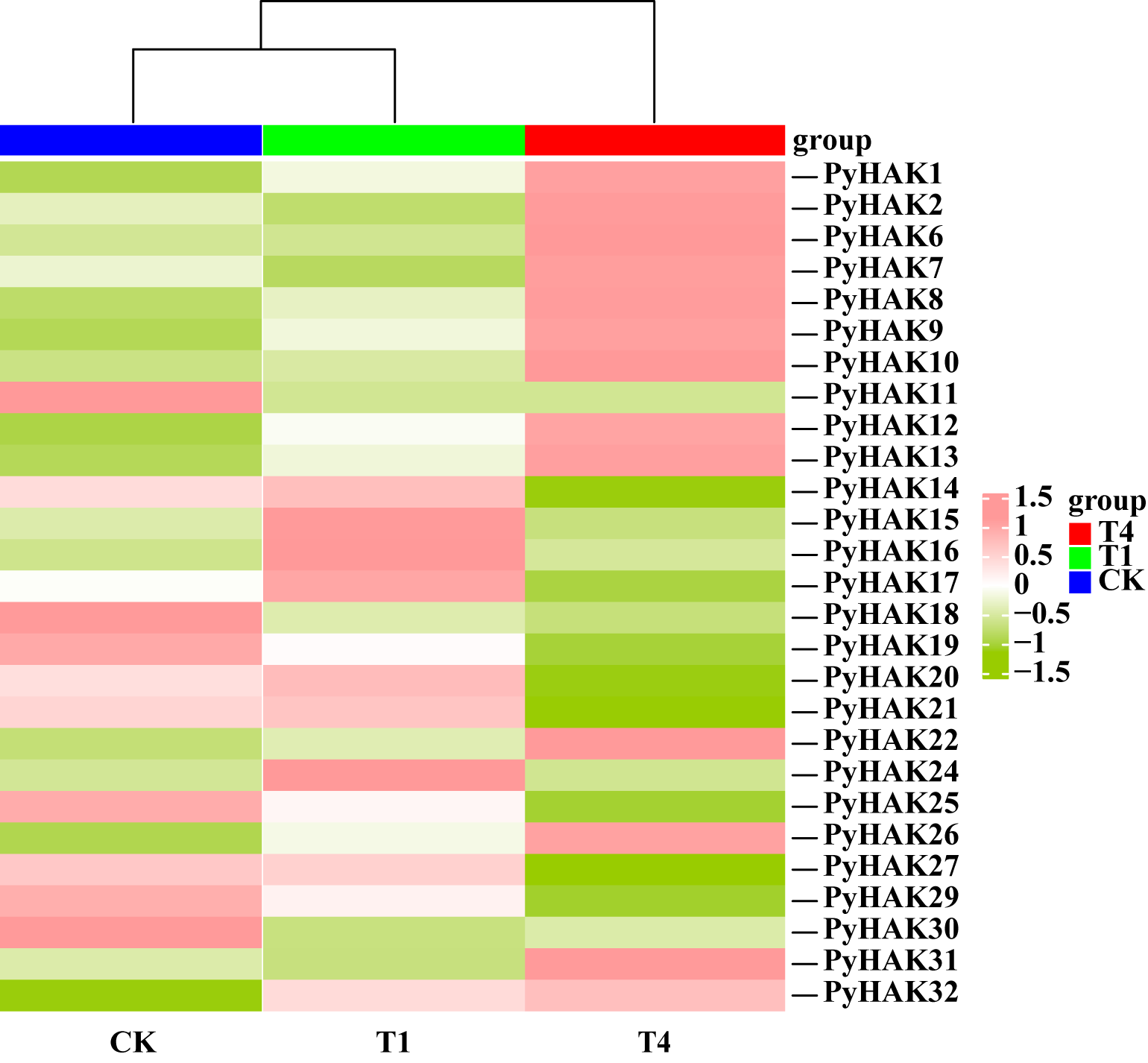

Supplement: Supplementary Figure 4 — Expression patterns of PyHAK genes based on RNA-seq analysis. [file Image4.tif]
